# Efficacy and Safety of Text Messages Targeting Adherence to Cardiovascular Medications in Secondary Prevention: TXT2HEART Colombia Randomized Controlled Trial

**DOI:** 10.2196/25548

**Published:** 2021-07-28

**Authors:** Anderson Bermon, Ana Fernanda Uribe, Paula Fernanda Pérez-Rivero, David Prieto-Merino, Jose Federico Saaibi, Federico Arturo Silva, Diana Ivonne Canon, Karol Melissa Castillo-Gonzalez, Diana Isabel Cáceres-Rivera, Elizabeth Guio, Karen Janneth Meneses-Castillo, Alberto Castillo-Meza, Louise Atkins, Robert Horne, Elizabeth Murray, Norma Cecilia Serrano, Caroline Free, Juan Pablo Casas, Pablo Perel

**Affiliations:** 1 Research Center Fundación Cardiovascular de Colombia Floridablanca Colombia; 2 Epidemiology an Biostatistics Escuela de Graduados Universidad CES Medellín Colombia; 3 Faculty of Psychology Universidad Pontificia Bolivariana - Seccional Bucaramanga Floridablanca Colombia; 4 Epidemiology and Population Health Faculty London School of Hygiene & Tropical Medicine London United Kingdom; 5 Applied Statistical Methods in Medical Research Group Universidad Católica San Antonio de Murcia Murcia Spain; 6 Departament of Cardiovascular Surgery Division of Vascular and Endovascular Surgery Fundación Cardiovascular de Colombia Floridablanca Colombia; 7 Neurovascular Science Group Fundación Cardiovascular de Colombia Floridablanca Colombia; 8 Departament of Cardiology Fundación Cardiovascular de Colombia Floridablanca Colombia; 9 Nursing Faculty Universidad Cooperativa de Colombia Bucaramanga Colombia; 10 Metabolism and Genoma Laboratory Fundación Cardiovascular de Colombia Floridablanca Colombia; 11 Research Department of Epidemiology and Public Health University College London London United Kingdom; 12 University College London School of Pharmacy London Colombia; 13 Research Department of Primary Care and Population Health University College London London United Kingdom; 14 Direction of Research Fundación Cardiovascular de Colombia Floridablanca Colombia; 15 Massachusetts Veterans Epidemiology Research and Information Center (MAVERIC) Boston, MA United States; 16 Department of Medicine, Brigham and Women's Hospital Harvard Medical School Boston, MA United States; 17 Centre for Global Chronic Conditions London School of Hygiene & Tropical Medicine London United Kingdom

**Keywords:** randomized controlled trial, Colombia, text messaging, cardiovascular disease, secondary prevention

## Abstract

**Background:**

Atherosclerotic cardiovascular disease (ASCVD) is the leading cause of mortality worldwide, with a prevalence of approximately 100 million patients. There is evidence that antiplatelet agents and antihypertensive medications could reduce the risk of new vascular events in this population; however, treatment adherence is very low. An SMS text messaging intervention was recently developed based on behavior change techniques to increase adherence to pharmacological treatment among patients with a history of ASCVD.

**Objective:**

This study aims to evaluate the efficacy and safety of an SMS text messaging intervention to improve adherence to cardiovascular medications in patients with ASCVD.

**Methods:**

A randomized controlled clinical trial for patients with a prior diagnosis of cardiovascular events, such as acute myocardial infarction, unstable angina, cerebrovascular disease, or peripheral artery disease, in one center in Colombia was conducted. Patients randomized to the intervention arm were assigned to receive SMS text messages daily for the first 4 weeks, 5 SMS text messages on week 5, 3 SMS text messages each in weeks 6 and 7, and 1 SMS text message weekly from week 8 until week 52. In contrast, patients in the control arm received a monthly SMS text message reminding them of the next study appointment and the importance of the study, requesting information about changes in their phone number, and thanking them for participating in the study. The primary endpoint was the change in low-density lipoprotein cholesterol (LDL-C) levels, whereas the secondary endpoints were the changes in thromboxane B2 levels, heart rate, systolic and diastolic blood pressure, medication adherence, cardiac and noncardiac mortality, and hospitalization. Linear regression analyses and bivariate tests were performed.

**Results:**

Of the 930 randomized patients, 805 (86.5%) completed follow-up and were analyzed for the primary endpoint. There was no evidence that the intervention changed the primary outcome (LDL-C levels; *P*=.41) or any of the secondary outcomes evaluated (all *P*>.05). There was also no evidence that the intervention was associated with adverse events.

**Conclusions:**

In this study, there was no evidence that a behavior modification intervention delivered by SMS text messaging improved LDL-C levels, blood pressure levels, or adherence at 12 months. More research is needed to evaluate whether different SMS text messaging strategies, including personalized messages and different timings, are effective; future studies should include mixed methods to better understand why, for whom, and in which context (eg, health system or social environment) SMS text messaging interventions work (or not) to improve adherence in patients with ASCVD.

**Trial Registration:**

ClinicalTrials.gov NCT03098186; https://clinicaltrials.gov/ct2/show/NCT03098186

**International Registered Report Identifier (IRRID):**

RR2-10.1136/bmjopen-2018-028017

## Introduction

### Background

Cardiovascular diseases are the leading cause of mortality worldwide. In 2017, approximately 17.5 million people died from cardiovascular diseases. Atherosclerotic cardiovascular disease (ASCVD) was responsible for 7.3 million deaths in 2007, which increased to 8.93 million in 2017. During the same period, mortality associated with cerebrovascular disease increased from 5.29 to 6.17 million events. Moreover, 82% of deaths in people ≤70 years occurred in low- and middle-income countries [1].

In 2015, more than 100 million people worldwide were diagnosed with ASCVD [2]. This population has been estimated to have a four- to five-fold increased risk of a new cardiovascular event in comparison with individuals without ASCVD history [3].

Robust evidence indicates that the use of antiplatelet agents, β-blocker agents, angiotensin-converting enzyme inhibitors (ACEIs), and statins reduces the incidence of fatal and nonfatal cardiovascular events in this population and is cost-effective. These medications are recommended by all international guidelines for the management of ASCVD [4,5].

However, long-term adherence to medication regimens continues to be suboptimal, and many patients stop medication for various reasons other than adverse side effects [6,7]. Only less than half of the patients with known ASCVD in high-income countries are receiving this group of cardiovascular medications, and the situation is much worse in low- to middle-income countries (LMICs), where only 1 in 20 patients with ASCVD received all four types of cardiovascular drugs in 2011 [8].

The widespread use of mobile devices allows the implementation of strategies such as text messaging to increase medication adherence. It has shown some promising results among patients with diabetes [9], HIV [10], and tuberculosis [11] and may therefore help improve adherence for patients with ASCVD [12,13]. In addition, access to the use of mobile telephones globally has increased in recent years. For example, in Colombia, telephone coverage increased from 84% in 2009 to 98.1% in 2019 [14].

A 2017 Cochrane review [15] evaluated the effects of SMS text messaging on medication adherence in patients with ASCVD. The review included 7 trials (n=1310) and reported the beneficial effect of SMS text messaging on adherence to medications in 6 of these trials. However, the quality of the evidence was very low. The Cochrane review identified the following limitations: (1) trials had small sample sizes (n=34-521); (2) most trials had a short follow-up period (<6 months); (3) the primary outcomes reported were of limited clinical relevance; (4) most studies recruited only patients with acute coronary syndrome and excluded an important group of patients with other arterial occlusive events (eg, stroke, peripheral vascular disease, and programmed coronary revascularization) who should be amenable for this type of intervention; (5) few studies were performed in LMICs; and (6) most trials did not describe the processes for SMS text messaging content generation, and the few trials that did report these processes did not target the key knowledge and attitudinal factors that are known to influence adherence to medication; instead, the interventions were simple *reminders* [15]. In summary, although there are some promising small studies, there is a need to provide high-quality evidence to assess the effect of SMS text messaging using behavior change techniques to increase long-term medication adherence in patients with ASCVD in LMICs.

### Aims

This study aims to fill this gap and provide evidence on whether theory-based and context-specific SMS text messages increase medication adherence for the secondary prevention of ASCVD in Colombia. We developed an intervention (ie, SMS text message) following the recommendations of Abroms et al [16]: a review of the literature, conduct of qualitative studies, and use of formal theories and behavior change techniques (Transtheoretical Model of Behavior Change). Details of our intervention development have been described previously [17,18].

The main aim of this study is to evaluate the efficacy and safety of an SMS text messaging intervention delivered by mobile phones to improve adherence to cardiovascular medications in patients with ASCVD. The intervention efficacy was assessed by measuring blood serum low-density lipoprotein cholesterol (LDL-C) levels as an indicator of adherence to statins, systolic blood pressure (SBP) as an indicator of adherence to blood-lowering therapies (ACEI or angiotensin II receptor blockers [ARBs]), and heart rate (HR) as an indication of adherence to β-blockers. The secondary objectives are to assess the impact of SMS text messaging on self-reported adherence to medications, hospitalizations, and the composite end point of incident major adverse cardiovascular events at 12 months.

## Methods

The full methodology of TXT2HEART Colombia has been previously published [17] and is summarized here. We report the following CONSORT (Consolidated Standards of Reporting Trials) recommendations [19].

### Study Design and Participants

In this two-arm parallel, single-blind individually randomized controlled trial, adult patients aged ≥18 years with a history of at least one of the following arterial occlusive events were included: acute coronary syndrome (unstable angina or acute myocardial infarction with or without ST elevation), stable angina, ischemic cerebrovascular disease, peripheral arterial disease, or coronary revascularization (coronary artery bypass surgery or percutaneous transluminal coronary angioplasty). Patients had to own a mobile phone and were able to read the SMS text message. They were excluded if they had a known contraindication to take all appropriate cardiovascular secondary prevention medications. All patients were recruited from a single center, the Fundación Cardiovascular de Colombia, a tertiary hospital serving as a reference center for cardiovascular diseases in Northeastern Colombia. The hospital has a clinical studies office and has been certified in good clinical practice by national and international authorities. All electronic health records were scanned using SQL queries, identifying patients with at least one month and without a maximum limit of time elapsed since the last hospitalization for ASCVD. The records were then manually inspected by 2 experienced medical doctors. Qualifying patients were contacted by phone, and if they met the inclusion criteria and were currently admitted or attended the outpatient clinic with a diagnosis of ASCVD, they were invited to participate. The process for evaluating potentially eligible individuals is described in Multimedia Appendix 1. Written informed consent was obtained from all subjects before the study.

### Intervention

The intervention consisted of behavior modification techniques based on the Transtheoretical Model [16] to be delivered via SMS text messaging. In our previous study [18], a protocol was carried out to determine the content, quantity, and frequency of SMS text messages through focus groups, validation of experts, user feedback, and pretest. The messages included information on the health implications of adherence to health habits (or lack thereof) and indications and recommendations on how to take their medication and promote healthy medication habits. They provided or encouraged social support activities for correct compliance with the prescribed treatment. The result of that study was 86 SMS text messages (including 12 SMS text messages of control and one welcome to the study), which were the messages used as an intervention in this clinical trial and the methodology of its delivery.

SMS text messages were sent through an automated text messaging platform (Telerivet), which was fed directly with data registered in Commcare, the platform for patient registration. Volunteers were informed about the unidirectional nature of the text messages and warned that no replies were expected. If the patients replied the word “PARE” or “Detener” (stop in Spanish), then no more messages were delivered. Text messaging was started a day after patient randomization. Messages were delivered every day for the first 4 weeks, and then five messages were delivered in week 5. From week 6 onward, three messages were delivered per week; from week 8 until week 52, one message was delivered per week. Messages were delivered on random weekdays from 8 AM to 6 PM to prevent patients from predicting delivery times, in accordance with a previous validation with study subjects. If the patient withdrew from the study or died, we stopped sending the SMS text messages. No tailoring considerations or modifications were made during the trial.

### Control Group

Patients in the control group only received SMS text messages regarding the next study appointment, requesting information about changes in their phone number, acknowledging them for participating in the study, and reminding them of the importance of the study. Messages were sent every month. These messages were also sent to the intervention group and were generated during SMS text message validation in the general population.

Examples of TXT2HEART Colombia SMS text messages are included in Multimedia Appendix 2.

### Outcomes

The primary outcome was a change in plasma LDL-C levels at 12 months. Blood samples were obtained at the start and end of the study appointment. An improvement in LDL-C levels was considered a surrogate indicator of adherence to statin treatment. The secondary outcomes were SBP as an indicator of adherence to blood-lowering therapies (ACEI or ARBs), HR as an indicator of adherence to β-blockers, 11-dehydrothromboxaneB2 urine levels adjusted for creatinine as an indicator of adherence to antiplatelet therapy, self-reported adherence to cardiovascular medications used in secondary prevention as measured using the Medication Adherence Report Scale-5 (MARS-5) questionnaire, and rates of cardiovascular death or hospitalization due to cardiovascular disease and noncardiovascular death or hospitalization due to noncardiovascular disease. We also included road traffic crashes (the only potential known hazard of SMS text messaging) and death due to all causes as secondary outcomes.

The psychometric properties of the MARS-5 have been previously reported [20]. The MARS-5 demonstrated acceptable reliability (internal and test-retest) and validity (criterion-related and construct validity). Internal reliability (Cronbach α) ranged from .67 to .89 across all patient groups; the test-retest reliability (Pearson *r*) was 0.97 for hypertension. Criterion-related validity was established with more adherent patients with hypertension showing better blood pressure control (*χ*^2^_1_=4.2; *P*=.04). Construct validity with beliefs about medicines was demonstrated; higher adherence was associated with stronger beliefs in treatment necessity and lower concerns about the medication.

All study participants were seen twice upon admission to the study for baseline assessment and randomization and at the end of the follow-up period for a 12-month office visit. A follow-up telephone call was made 3 months after randomization asking about new hospital admissions, all-cause death, or cardiovascular death, and adverse events were also recorded. SBP, resting HR, and urinary levels of thromboxane B2 were recorded at the first and final visits. Self-informed cardiovascular medication prescription compliance was assessed using the MARS-5 at both visits. The scale was applied by trained personnel, considering automatic compliance if a total score of 25 was achieved. Subjective medication intake compliance was assessed on days 7 and 30. Data obtained about recurrent ASCVD were requested on the phone interview or by physical examination; oral reports by patients or relatives were allowed; and cardiovascular or any cause mortality was recorded. Information obtained on the phone was confirmed in all cases by reviewing medical case notes, registries, or death certificates. Written evidence for any event was requested via electronic mail, WhatsApp messaging, or case note copies. If death or any major event or hospital admission occurred during the follow-up period, a hard copy of the death certificate from the patients’ relatives or case notes was requested on the 12-month follow-up visit. At the final follow-up, all biomarkers were processed simultaneously to avoid interference due to the reactive processing, and the simple handling protocol depicted in the protocol was followed [17]. At the initial appointment, we recorded at least three different phone numbers and a complete house address for each subject. A study identification card was provided with a written record of the date of the last visit to the trial, name of the principal investigator, and clinic contact phone numbers. To ensure no loss of follow-up, any home-number modifications were actively searched and recorded. The appointment follow-up interval was kept to avoid any interference with the study results.

### Sample Size

The original sample size of the study was 1600 participants, based on a 97% power to detect a 10% difference between arms in adherence. In the published protocol [17] of this trial, a table with power calculations under different assumptions was included. However, due to limited study funding, the final sample recruited was 930 participants, 805 (86.5%) of whom reported LDL-C at the final visit. With 400 patients per arm (based on an expected mean reduction of 80 mg/dl on plasma levels of LDL-C in an adherence population and an expected mean reduction of 16 mg/dl on a nonadherent population to atorvastatin 20 mg) and assuming a 5% type I error, a power >92% was estimated to detect a 10% difference in protocol compliance between both intervention arms. A table with power calculations for this sample size under different assumptions is included in Multimedia Appendix 3.

### Randomization

Block randomization was used, with block sizes of 5 patients each in a 1:1 allocation ratio, and assignment was done automatically using a remote computer-based randomization. Once the patient met the inclusion and exclusion criteria and signed the consent form, the data capture platform Commcare (Dimagi) applied a logarithm of randomization assigning the arm for the patient. This information was not shown to the interviewer to maintain blindness, although he did confirm its effective completion on the digital form. The data capture platform accessed the services of the SMS text messaging platform (Telerivet), categorizing the SMS text messaging group to send according to the assigned group.

### Blinding

Owing to the nature of the intervention, the participants could not be blinded. However, all investigation personnel inputting data were blinded to the individual’s group assignment, and all patients were asked not to reveal their allocation details to the study personnel. The study had an engineer who was the only person who could access the messaging and database platforms. He could access the data to sort patient queries or help solve reception or technical issues. He was specifically trained on the importance of maintaining blinding. Investigators handling and analyzing the data were blinded to the intervention assigned.

### Statistical Analysis

The distributions of the baseline characteristics were compared between the intervention and control groups for all randomized patients, those who completed the follow-up and those who did not complete the follow-up, performing an intention to treat analysis. Analysis of the continuous outcomes (ie, LDL-C, thromboxane, HR, SBP, diastolic blood pressure [DBP], and quantitative measures of adherence) was performed using linear regression models. In each model, the dependent variable was the difference between 12-month follow-up and the baseline of the outcome, and the main explanatory variable was the intervention group. Furthermore, the Patient Health Questionnaire-9 (PHQ-9) scale (to measure depression) was collected at baseline, as it was considered a potential confounding factor for adherence to medication. All linear regression models were adjusted by the baseline value of the outcome, centered on the mean. The effect of the intervention was the difference between the arms in the expected change in an individual and the average outcome value at baseline. Binary outcomes (ie, hospitalization and mortality) were analyzed by comparing the proportion of occurrence in both arms. No adverse events were reported; therefore, we did not conduct any analyses regarding this outcome. All *P* values were from two-sided tests, and the data were analyzed using Stata version 14.0 (StataCorp). No interim analyses were performed.

### Ethical Considerations

The Ethics Committee of the Fundación Cardiovascular de Colombia evaluated and approved the trial (reference 375-2015). The study was conducted in compliance with the protocol, regulatory requirements, Good Clinical Practice, the Declaration of Helsinki, and the clinical investigations guidelines of the Fundación Cardiovascular de Colombia.

### Data Availability

The data sets generated during this study are not publicly available because availability was not included in the study plan approved by the ethics committee but are available from the corresponding author on reasonable request.

## Results

### Participant Flow

**Figure 1:**
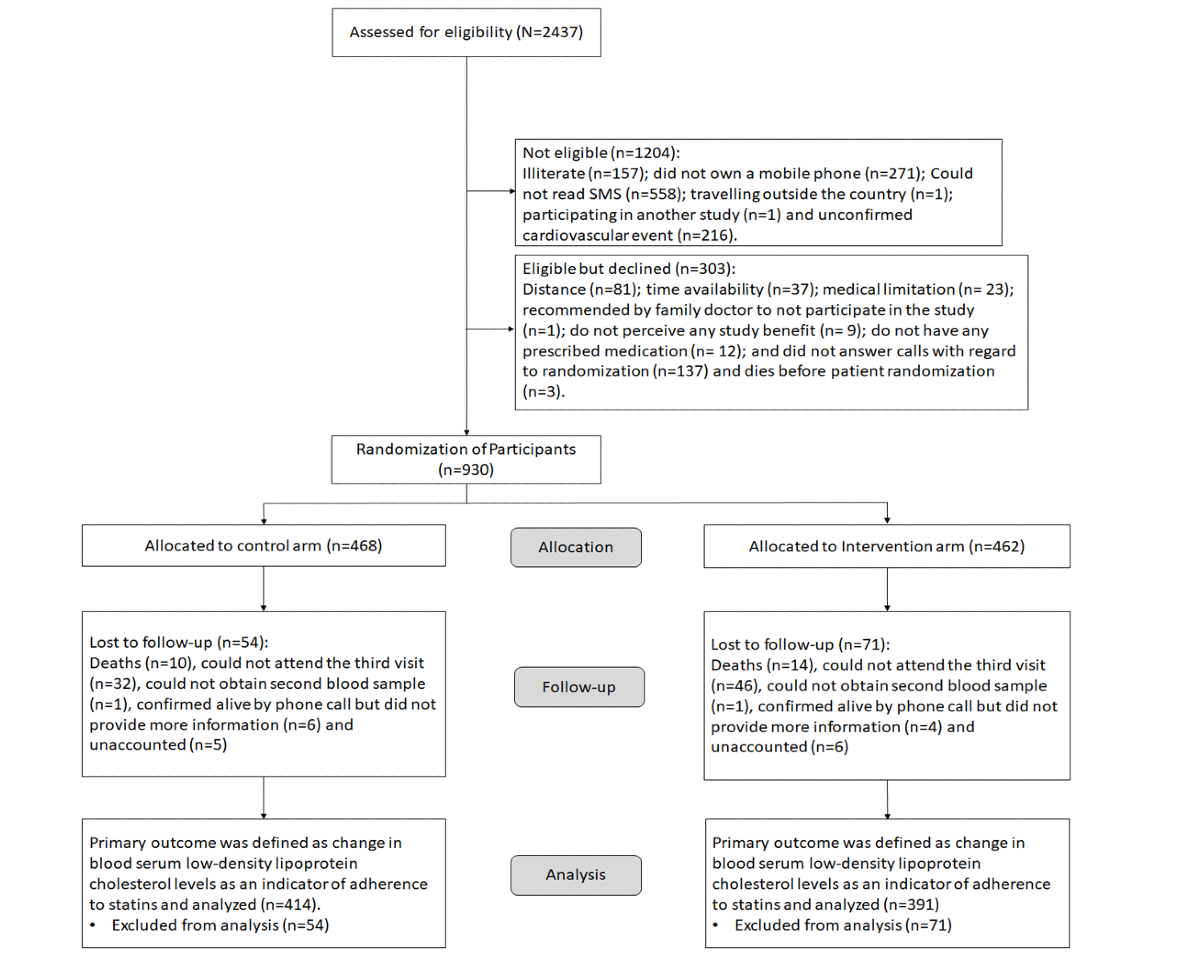
CONSORT (Consolidated Standards of Reporting Trials) diagram.

From April 18, 2017, to August 21, 2018, 930 patients were randomized. A total of 49.7% (462/930) of patients were assigned to the intervention arm and 50.3% (468/930) were included in the control arm (Figure 1), of which 1.7% (16/930) of patients replied “stop” to the messages (6 in intervention group and 10 control), all of whom were followed up until the end of the study. In total, 13.4% (125/930) losses to follow-up occurred, 71 in the intervention group and 54 in the control group.

A total of 86.8% (805/930) of participants completed the trial follow-up at 12 months for the primary outcome (intervention group: n=391; control: n=414; Figure 1). Retention did not differ between two arms (71/462, 15.4% in the intervention group vs 54/468, 11.5% in the control group; *P*=.09). The main predictors of retention were male sex (OR 1.61, 95% CI 1.05-2.46; *P*=.03) and high total PHQ-9 score (OR 0.37, 95% CI 0.15-0.92; *P*=.03). The effect of these predictors did not differ between the groups (interaction test values: *P=*.23 and *P*=.80, respectively). The characteristics of the participants who completed the follow-up and those who did not are reported in Multimedia Appendix 4. For secondary outcomes, HR, SBP, and DBP of 850 patients were evaluated. For TxBA2, only 801 patients were evaluated because 4 patients were unable to deliver the urine sample. In addition, 807 patients were assessed using the MARS-5. New cardiovascular events were evaluated in 910 patients and mortality was evaluated in 919 patients through telephone interviews.

### Baseline Characteristics

**Figure 2:**
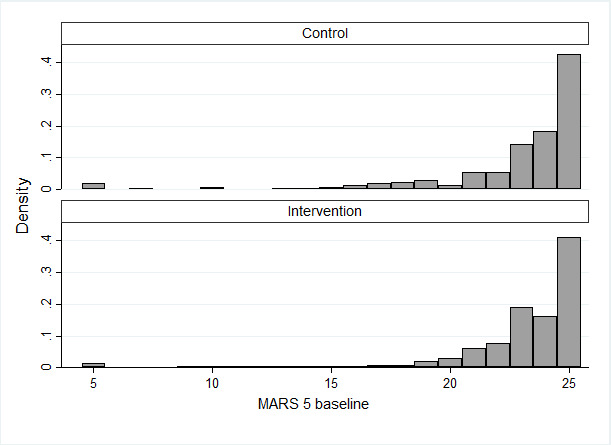
Distribution of the MARS-5 scores at baseline. Graphs by Intervention. MARS-5: Medication Adherence Report Scale-5.

The baseline characteristics of the participants (N=930), which were similar between the two groups, are summarized in Table 1. Overall, most participants (729/930, 78.4%) were men, and the mean age was 63.5 years (SD 9.8); 88.9% (827/930) were using statins, 94.52% (879/930) were on antiplatelet aggregation therapy, and β-blocker use was reported in 83.2% (774/930) of participants, whereas ACEI or ARBs use was observed in 68.1% (633/930) of participants. The average MARS-5 score at baseline was 22.8, and 41.72% (388/930) of participants considered adherent (MARS-5 score=25). Clinical and laboratory characteristics such as PHQ-9, BMI, LDL-C, SBP, DBP, HR, and thromboxane B2 were similar between the two groups (Table 1). The baseline characteristics of participants with primary outcome completers and primary outcome noncompleters are shown in Multimedia Appendix 4, and the MARS-5 scores at baseline are shown in Figure 2.

**Table 1:** Characteristics of study participants at baseline (N=930).

Characteristics			Control group (n=468)		Intervention group (n=462)		Participants (N=930)
Age (years), mean (SD)			63.1 (10)		64.0 (9.7)		63.5 (9.8)
**Gender, n (%)**							
	Female	92 (19.7)		109 (23.6)		201 (21.6)	
	Male	376 (80.3)		353 (76.4)		729 (78.4)	
**Time since the last event, n (%)**							
	Less than 3 months	62 (13.3)		43 (9.3)		105 (11.3)	
	3 to 12 months	84 (17.9)		97 (21)		181 (19.5)	
	1 to 3 years	144 (30.8)		122 (26.4)		266 (28.6)	
	More than 3 years	178 (38)		200 (43.3)		378 (40.7)	
**Type event, n (%)**							
	Acute coronary syndrome	336 (71.8)		327 (70.8)		663 (71.3)	
	Stable angina	33 (7.1)		23 (5)		56 (6)	
	Ischemic cerebrovascular disease	21 (4.5)		21 (4.6)		42 (4.5)	
	Peripheral arterial disease	15 (3.2)		21 (4.6)		36 (3.9)	
	Coronary revascularization	63 (13.5)		70 (15.2)		133 (14.3)	
**Prescribed medications, n (%)**							
	Statins	414 (88.5)		413 (89.4)		827 (88.9)	
	ACEI^a^ or ARBs^b^	327 (69.9)		306 (66.2)		633 (68.1)	
	β-blocker	382 (81.6)		392 (84.9)		774 (83.2)	
	Platelet aggregation inhibitors	442 (94.4)		437 (94.6)		879 (94.5)	
MARS-5^c^ score, mean (SD)			22.8 (3.78)		23 (3.31)		22.9 (3.6)
Adherent (MARS-5 score=25 points), n (%)			199 (42.5)		189 (40.9)		388 (41.7)
**Self-reported adherence, mean (SD)**							
	Last 7 days (0-10 scale)	9.1 (2.04)		9.1 (2.19)		9.1 (2.1)	
	30 days (0-10 scale)	9.1 (1.95)		9.1 (2.05)		9.1 (2)	
**PHQ-9^d^, n (%)**							
	Minimal depression (<5)	343 (73.3)		323 (69.9)		666 (71.6)	
	Moderate depression (5-14)	112 (24)		127 (27.5)		239 (25.7)	
	Moderately severe depression or severe (>14)	12 (2.6)		13 (2.8)		25 (2.7)	
**Smoking, n (%)**							
	Smoker	16 (3.4)		12 (2.6)		28 (3)	
	Never smoked	168 (35.9)		185 (40)		353 (38)	
	Past smoker	284 (60.7)		265 (57.4)		549 (59)	
BMI (m/kg^2^), mean (SD)			27.9 (4.2)		27.3 (4.2)		27.6 (4.2)
LDL^e^ (mg/dl), mean (SD)			88.2 (37.4)		88.5 (38.0)		88.4 (37.7)
SBP^f^ (mmHg), mean (SD)			128.2 (20.8)		129.9 (20.9)		129.0 (20.9)
DBP^g^ (mmHg), mean (SD)			71.5 (11.7)		71.9 (11.3)		71.7 (11.5)
Heart rate (bpm), mean (SD)			68.9 (11.7)		68.8 (10.6)		68.8 (11.1)
Thromboxane B2^h^ (ng/ml), mean (SD)			64.1 (147.2)		64.2 (167.7)		64.2 (157.6)

^a^ACEI: angiotensin-converting enzyme inhibitor.

^b^ARB: angiotensin II receptor blocker.

^c^MARS-5: Medication Adherence Report Scale-5.

^d^PHQ-9: Patient Health Questionnaire-9.

^e^LDL: low-density lipoprotein.

^f^SBP: systolic blood pressure.

^g^DBP: diastolic blood pressure.

^h^Creatinine adjusted (mg/dl).

### Outcomes

We did not find evidence (*P=*.41) that the intervention was more effective than the control according to changing plasma LDL-C levels (adjusted by baseline value) at 12 months. We also did not find significant differences between the two groups in terms of secondary outcomes analyzed including thromboxane B2 levels, HR, SBP, DBP, adherence measured by MARS-5, or clinical events (hospitalization or death) at one year of follow-up (Table 2).

There were no adverse events related to this study. In total, three falls that required hospitalization were reported; in all of them, medical evaluation and patient interviews were performed, eliminating any relationship between mobile phone use and fall events. No traffic accidents occurred.

**Table 2:** Summary of primary and secondary outcomes^a^.

Outcome		Baseline		Difference^b^			Difference adjusted by baseline			
		Control	Intervention	Control (n=468)	Intervention (n=462)	Coefficient (95% CI)		Odds ratio (95% CI)	Risk ratio (95% CI)	*P* value
**Primary outcome, mean (SD)**										
	LDL^c^ (mg/dl)	88.0 (36.9)	88.0 (37.5)	5.1 (31.8)	7.0 (33.8)	1.85 (−2.5 to 6.2)		N/A^d^	N/A	.42
**Secondary outcome**										
	Thromboxane B2 (ng/ml)^e^, mean (SD)	61.2 (133.2)	58.8 (138.1)	−19.6 (131.0)	−18.6 (94.0)	−0.28 (−10.54 to 10.0)		N/A	N/A	.96
	Heart rate (bpm), mean (SD)	68.6 (11.6)	68.5 (10.5)	−0.1 (13.9)	0.6 (10.5)	0.54 (−1.0 to 2.1)		N/A	N/A	.48
	SBP^f^ (mmHg), mean (SD)	128.0 (21.2)	129.3 (20.6)	1.3 (19.4)	0.8 (20.7)	0.14 (−2.3 to 2.6)		N/A	N/A	.91
	DBP^g^ (mmHg), mean (SD)	71.5 (11.9)	71.7 (11.3)	0.7 (11.7)	−0.1 (10.7)	−0.70 (−2.0 to 0.6)		N/A	N/A	.30
	MARS-5^h^ (score), mean (SD)	22.8 (3. 8)	23.1 (3.1)	0.2 (3.7)	−0.02 (3.4)	−0.01 (−0.4 to 0.4)		N/A	N/A	.96
	Self-reported adherence (7 days), mean (SD)	9.1 (2.1)	9.2 (2.0)	0.1 (2.0)	0.2 (2.0)	0.05 (−0.2 to 0.3)		N/A	N/A	.69
	Self-reported adherence (30 days), mean (SD)	9.1 (2.0)	9.2 (1.9)	0.1 (1.9)	0.1 (2.0)	0.02 (−0.2 to 0.2)		N/A	N/A	.83
	Change in adherence^i^, mean (SD)	N/A	N/A	1.2 (0.2)	1.1 (0.20)	N/A		0.94 (0.6 to 1.5)	N/A	.81
	Hospitalization for cardiovascular events^j^, n (%)	N/A	N/A	32 (6.8)	27 (5.8)	N/A		N/A	0.85 (0.5 to 1.4)	.54
	Hospitalization for any cause^k^, n (%)	N/A	N/A	49 (10.5)	50 (10.8)	N/A		N/A	1.03 (0.71 to 1.5)	.92
	Death from cardiovascular events^l^, n (%)	N/A	N/A	3 (0.6)	2 (0.4)	N/A		N/A	0.68 (0.1 to 4.0)	.99
	Death from any cause^j^, n (%)	N/A	N/A	8 (1.7)	14 (3)	N/A		N/A	1.80 (0.8 to 4.3)	.20

^a^Sample sizes may vary slightly because some individuals have missing values.

^b^Calculated as the difference between final and baseline measurements.

^c^LDL: low-density lipoprotein.

^d^N/A: not applicable.

^e^Creatinine adjusted (mg/dl)

^f^SBP: systolic blood pressure.

^g^DBP: diastolic blood pressure.

^h^MARS-5: Medication Adherence Report Scale-5.

^i^Within each group is the number of patients that lose adherence over those that became adherent.

^j^Within each group is the proportion of patients who experienced the event.

## Discussion

### Principal Findings

In this study, a behavioral modification intervention delivered by SMS text messages did not decrease LDL-C level, the primary outcome, and did not find evidence of an impact on any of the other biological markers assessed, including blood pressure, HR, or thromboxane; clinical events; or medication adherence, as measured by the MARS-5 or self-reporting.

This study has many strengths. First, a thorough and detailed formative research was followed to develop a tailored behavioral modification intervention, which has been previously published [17] alongside the study protocol. Different outcomes have been collected, including self-reporting of adherence, validated adherence tools (ie, the MARS-5 has demonstrated acceptable reliability [internal and test-retest] and validity [criterion-related and construct validity]) [20], and proxy biological markers, and they were triangulated to evaluate adherence. A rigorous plan was put in place to ensure that the intervention was delivered appropriately, study investigators collecting data were blinded to the patient allocation arm, and most patients were followed up for the primary and secondary outcomes at 12 months. Finally, even if the sample size was smaller than originally planned, to the best of our knowledge, this study is the largest to date to assess the effect of a behavioral modification intervention delivered by SMS text messages to increase adherence in people with ASCVD.

### Limitations

This study also presented some limitations; although sending text messages could be confirmed, neither the correct reading of the message nor the stage of the transtheoretical behavioral modification reached by each participant was evaluated [18]. In addition, the inability to blind patients would have increased the likelihood of underreporting nonadherence. This is a common problem with self-reports when patients may exaggerate their adherence if they believe that reports of nonadherence will disappoint their health provider (self-presentational bias) [21]. The MARS-5 addresses this problem by taking steps to diminish self-presentational bias. Introductory statements normalize nonadherence, conveying a *no-blame* approach [8]. As another step to minimize self-presentational bias, patients were told that their responses to the study questionnaires would not be seen by the health care professionals providing care. In addition, we conducted a case analysis on all lost cases, assuming that all related losses were random, but due to the low rate of loss to follow-up and that its main predictors (sex and PHQ-9) were similar in both arms of the study, we do not consider that these losses had a major impact on the results of the trial.

Another limitation was that SMS text messages were not customized according to the patient categories. However, as mentioned earlier, this formative research was detailed, and a formal theory was applied to develop the content of the messages. Unfortunately, due to the lack of funding, we could not conduct a comprehensive mixed methods process evaluation to shed light on some of the mechanisms and contexts that could explain the effect on specific subgroups of patients. Finally, measuring adherence is always challenging; a validated scale (ie, MARS-5) was used and self-reporting measures were used, but pill count was not taken into consideration; however, the direct measurement of adherence was complemented with indirect measurements, such as those for LDL-C, blood pressure, HR, and thromboxane.

A previous Cochrane review [15] published in 2017 identified studies reporting positive effects; however, all studies had small sample sizes and high risk of bias and therefore provided a low level of evidence. The TEXT ME study was a randomized controlled trial that included 710 patients with coronary artery disease who did not report medication adherence but did report that four text messages sent per week led to reductions in LDL-C levels and blood pressure at 6 months [22]; however, a more recent multi-center randomized controlled trial in China including 822 patients with coronary artery disease reported that SMS text messages did not reduce blood pressure or LDL-C levels at 6 months [23].

The lack of evidence of a beneficial effect reported by the intervention could be due to different reasons; the study might have lacked the power to detect an effect as the sample size was lower than intended; however, with 930 patients, it was well powered to detect a reasonable, modest, clinical benefit. The lack of benefit could be potentially explained by the fact that the baseline levels of medication adherence were already quite high, with a mean MARS-5 of 9.1 out of 10 at 7 and 30 days; therefore, there was little room for improvement. Another possible explanation could be related to intervention content. Although the intervention went beyond simple prompts and reminders (a thorough process was followed with the objective of changing beliefs and motivations), it was not tailored enough to change beliefs and motivations in the study context, or perhaps in this highly adherent population, these issues were not the main drivers of nonadherence. The findings could also be related to issues related to the delivery of the SMS text messages (ie, timing, frequency, and length of intervention), due to the effects of fatigue, overload, or loss of interest in SMS text messages. SMS text messaging interventions have been shown to be effective in modifying lifestyle issues (eg, tobacco cessation) where the goals of the patient and the intervention are closely aligned; however, they might be less effective on adherence [24]. Another possible explanation is that the results were measured at 12 months, and it has been shown that adherence interventions are more effective in the short term (3 to 6 months) [25]. Planning only one intermediate measure at 3 months to assess the presence of rehospitalizations or death may limit the comparison of this study with others, but the protocol prioritized the pragmatic conditions of the trial, avoiding face-to-face contacts (eg, adherence questions), because this would represent an additional study activity to the real scenario of the patients, leading to a potential Hawthorne effect that can alter the results of the effectiveness of SMS text messages. Another explanation for the difference found in the studies included in the Cochrane review could be related to the fact that only a third of the patients recruited in our study had an index event within the last year.

Finally, having data from a single site is a limitation in generalizing our results to different scenarios; however, it is important to note that the institution where the study was conducted is a reference center in Northeastern Colombia, and its area of influence included a population of 5 million.

### Conclusions

This study did not find evidence that a behavior modification intervention delivered by SMS text messaging improves medication adherence, LDL-C, or blood pressure levels at 12 months. Although its potential use to increase medication adherence in patients with ASCVD remains as a potentially attractive and scalable solution to a very important problem, further research is needed. Future research should include interventions, including SMS text messages blended with other components, tailored to the specific issues and beliefs of individual participants. Implementation studies using mixed methods and innovative approaches that evaluate how different intervention characteristics (behavioral modification component), SMS text message delivery strategies (eg, timing of initiation, frequency, duration, and personalization), and context (eg, type of patients, health system, and social environment) influence the effect of this intervention should be conducted.

## Appendix

Multimedia Appendix 1 Evaluating potential eligible individuals.

Multimedia Appendix 2 Example of the SMS text messaging intervention.

Multimedia Appendix 3 Sample size calculations.

Multimedia Appendix 4 Baseline characteristics.

Multimedia Appendix 5 CONSORT-eHEALTH checklist (v.1.6.1).

## Abbreviations

ACEI: angiotensin-converting enzyme inhibitor
ARB: angiotensin II receptor blocker
ASCVD: atherosclerotic cardiovascular disease
CONSORT: Consolidated Standards of Reporting Trials
DBP: diastolic blood pressure
HR: heart rate
LDL-C: low-density lipoprotein cholesterol
LMIC: low- to middle-income country
MARS-5: Medication Adherence Report Scale-5
PHQ-9: Patient Health Questionnaire-9
SBP: systolic blood pressure
