# Supplementary figures and images for: Efficacy and Safety of Text Messages Targeting Adherence to Cardiovascular Medications in Secondary Prevention: TXT2HEART Colombia Randomized Controlled Trial
Source: JMIR Mhealth Uhealth. 2021 Jul 28;9(7):e25548. doi: 10.2196/25548 (PMC8367158; doi:10.2196/25548)

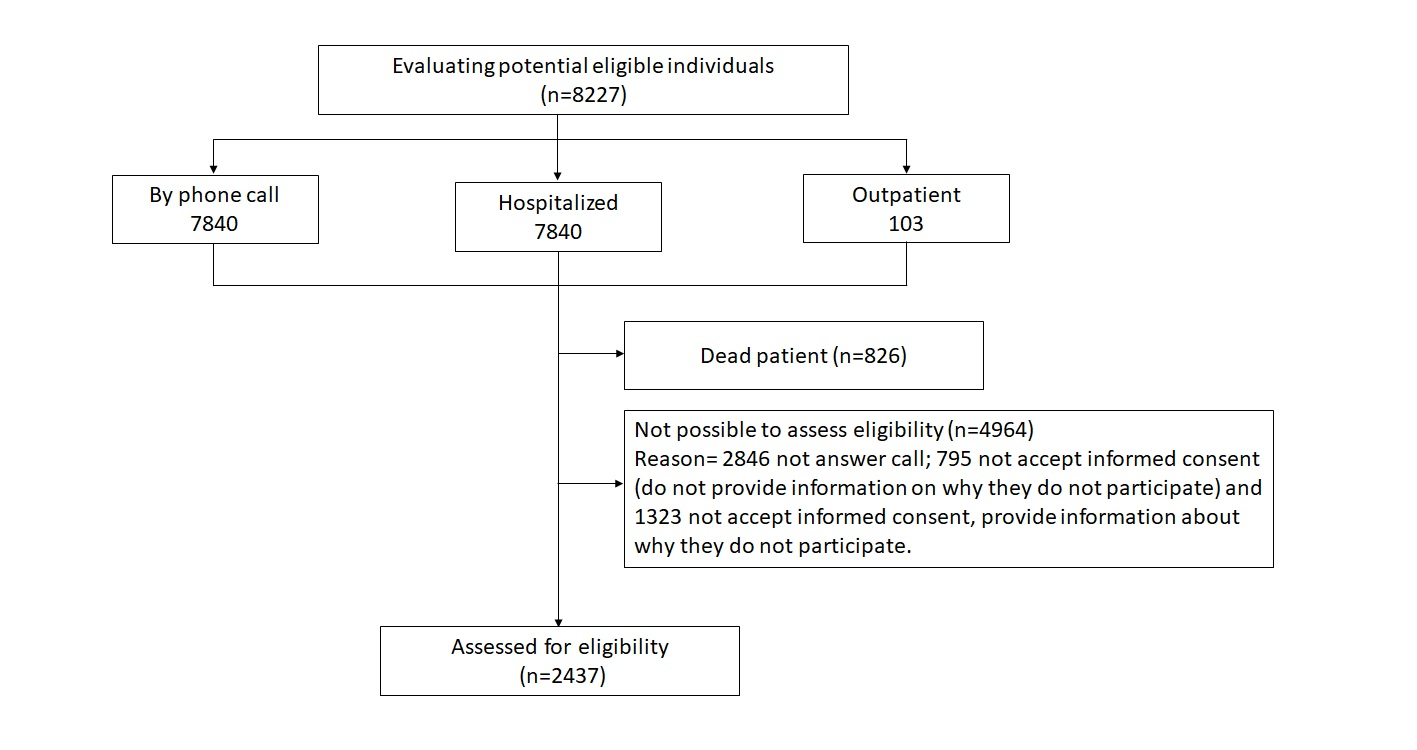

Supplement: Multimedia Appendix 1 [file mhealth_v9i7e25548_app1.png]
