# Supplementary material for: Efficacy and Safety of Text Messages Targeting Adherence to Cardiovascular Medications in Secondary Prevention: TXT2HEART Colombia Randomized Controlled Trial
Source: JMIR Mhealth Uhealth. 2021 Jul 28;9(7):e25548. doi: 10.2196/25548 (PMC8367158; doi:10.2196/25548)
Supplement: Multimedia Appendix 2 [file mhealth_v9i7e25548_app2.docx]

Appendix 2

| Example of the SMS intervention:  “Un tratamiento farmacológico adecuado le podría ayudar a prevenir la aparición de nuevos eventos cardiovasculares”  (An adequate medication will help prevent new cardiovascular events )  “Un pastillero es una cajita donde puede organizar los medicamentos para tomar durante el día y la semana, son de mucha ayuda. Se consiguen en las farmacias”  (A pill organizer is helpful for organizing your daily or weekly medication, you can find them at any pharmacy)  Control group SMS text:  “Gracias por su participación en el estudio "Txt2Heart Colombia", recuerde hacernos saber si sus datos de contacto han cambiado al teléfono…”.  (We appreciate your participation in the "Txt2Heart Colombia", investigation, do not forget to inform on this same phone number us if your contact data has changed ) |
| --- |
