# Supplementary material for: Efficacy and Safety of Text Messages Targeting Adherence to Cardiovascular Medications in Secondary Prevention: TXT2HEART Colombia Randomized Controlled Trial
Source: JMIR Mhealth Uhealth. 2021 Jul 28;9(7):e25548. doi: 10.2196/25548 (PMC8367158; doi:10.2196/25548)
Supplement: Multimedia Appendix 3 [file mhealth_v9i7e25548_app3.docx]

Appendix 3.

| Sample size calculations | |  |  | | |  |  | | |
| --- | --- | --- | --- | --- | --- | --- | --- | --- | --- |
| Statins and its fequency in trials | |  | Reduction in LDL after a year of treatment in adherents and non-adherents | | |  | Power to detect differences depending on adherence increase | | |
|  | % |  | AD=yes | AD No | Dif |  | 5.0% | 7.0% | 10.0% |
| Atorvastatin 10 | 1.5% |  | 69.2 | 13.8 | 55.4 |  | 30% | 53% | 82% |
| Atorvastatin 20 | 32.9% |  | 80.0 | 16.0 | 64.0 |  | 39% | 65% | 92% |
| Atorvastatin 40 | 52.4% |  | 91.3 | 18.3 | 73.0 |  | 48% | 76% | 97% |
| Atorvastatin 80 | 9.4% |  | 102.1 | 20.4 | 81.7 |  | 57% | 85% | 99% |
| Fluvastatin 20 | 0.0% |  | 39.4 | 7.9 | 31.6 |  | 13% | 21% | 38% |
| Lovastatin 40 | 0.0% |  | 68.4 | 13.7 | 54.8 |  | 30% | 52% | 82% |
| Pravastatin 10 | 0.0% |  | 36.7 | 7.3 | 29.4 |  | 12% | 19% | 34% |
| Pravastatin 20 | 0.0% |  | 45.2 | 9.0 | 36.2 |  | 16% | 26% | 47% |
| Pravastatin 40 | 0.0% |  | 53.4 | 10.7 | 42.7 |  | 20% | 35% | 61% |
| Rosuvastatin 5 | 0.0% |  | 71.2 | 14.2 | 56.9 |  | 32% | 55% | 84% |
| Rosuvastatin 10 | 0.3% |  | 80.4 | 16.1 | 64.3 |  | 39% | 65% | 92% |
| Rosuvastatin 20 | 1.7% |  | 89.7 | 17.9 | 71.8 |  | 47% | 75% | 96% |
| Rosuvastatin 40 | 1.9% |  | 99.0 | 19.8 | 79.2 |  | 54% | 83% | 99% |
| Simvastatin 10 | 0.0% |  | 50.7 | 10.1 | 40.5 |  | 18% | 32% | 56% |
| Simvastatin 20 | 0.1% |  | 59.6 | 11.9 | 47.6 |  | 24% | 41% | 70% |
| Simvastatin 40 | 0.2% |  | 68.8 | 13.8 | 55.1 |  | 30% | 52% | 82% |
| Simvastatin 80 | 0.0% |  | 77.7 | 15.5 | 62.2 |  | 37% | 62% | 90% |

Power is calculated assuming a sample size of 800 a type-I error of 5%, a standard deviation of the effect of the statins on the reduction of LDL-C of 27.07 mg/dl, and that non-adherent patients will have an effect of reduction of LDL-C of 20% of the effect in adherent patients.

Interpretation of the table: example of second line (Atorvastatin 20mg/daily for 12 months) there will be, on average, a reduction of LDL-C of 80.0 mg/dL, on the contrary a non-adherent patient will only reduce LDL-C by 16.0 mg/dL (about 20% of the reduction in the adherent patient). Thus If all patients in the trial received atorvastatin 20mg/day and the efficacy of the SMS to improve adherence is of 10%, we will expect that in the intervention arm the reduction of LDL-C will be on average 6.4 mg/dl greater than in the control arm and we will have a 92% power to detect this difference

*Elaborated by the authors

LDL, low-density lipoprotein
