# Supplementary material for: Efficacy and Safety of Text Messages Targeting Adherence to Cardiovascular Medications in Secondary Prevention: TXT2HEART Colombia Randomized Controlled Trial
Source: JMIR Mhealth Uhealth. 2021 Jul 28;9(7):e25548. doi: 10.2196/25548 (PMC8367158; doi:10.2196/25548)
Supplement: Multimedia Appendix 4 [file mhealth_v9i7e25548_app4.docx]

Appendix 4

| Baseline characteristics | | Primary outcome completers | | | Primary outcome non-completers | | | | | |
| --- | --- | --- | --- | --- | --- | --- | --- | --- | --- | --- |
|  |  | Control  N= 414 | Intervention N=391 | All participants N=805 | Control  N= 54 | | Intervention N=71 | | All participants N=125 | |
| Age, mean [SD] | | 62.7 [0.5] | 63.9 [0.5] | 63.3 [0.4] | 65.5 [11.0] | | 64.5 [8.1] | | 65.0 [9.5] | |
| Gender, female, n (%) | | 78 (18.8) | 87 (22.3) | 165 (20.5) | 14 (25.9) | | 22 (31.0) | | 36 (28.8) | |
| **Time since the last event, n (%)** | |  |  |  |  | |  | |  | |
|  | Less than 3 months | 53 (12.8) | 37 (9.5) | 90 (11.2) | 9 (16.7) | | 6 (8.5) | | 15 (12.0) | |
|  | 3 to 12 months | 71 (17.2) | 84 (21.5) | 155 (19.3) | 13 (24.1) | | 13 (18.3) | | 26 (20.8) | |
|  | 1 to 3 years | 131 (31.6) | 102 (26.1) | 233 (28.9) | 13 (24.1) | | 20 (28.2) | | 33 (26.4) | |
|  | More than 3 years | 159 (38.4) | 168 (43.0) | 327 (40.6) | 19 (35.2) | | 32 (45.1) | | 51 (40.8) | |
| **Prescribed with** | |  |  |  |  | |  | |  | |
|  | Statins | 368 (88.9) | 354 (90.5) | 722 (89.7) | 46 (85.2) | | 71 (83.1) | | 105 (84) | |
|  | ACEi or ARBs | 289 (69.8) | 256 (65.5) | 545 (67.7) | 38 (70.4) | | 50 (70.4) | | 88 (70.4) | |
|  | BB | 334 (80.7) | 334 (85.4) | 668 (83.0) | 48 (88.9) | | 58 (81.7) | | 106 (84.8) | |
|  | Platelet aggregation inhibitors | 392 (94.7) | 371 (94.9) | 763 (94.8) | 50 (92.6) | | 66 (93.0) | | 116 (92.8) | |
| MARS, mean [SD] | | 22.8 [0.2] | 23.1 [0.16] | 22.9 [0.3] | 22.8 [3.8] | | 22.3 [4.2] | | 22.5 [4.02] | |
| Adherent (MARS=25 points), n (%) | | 175 (42.3) | 161 (41.2) | 336 (41.7) | 24 (44.4) | | 28 (39.4) | | 52 (41.6) | |
| Self-reported adherence, last 7 days, (0-10 scale), mean [SD] | | 9.1 [0.1] | 9.2 [0.1] | 9.1 [0.1] | 9.2 [1.5] | | 8.5 [2.9] | | 8.8 [2.4] | |
| Self-reported adherence, 30 days, (0-10 scale), mean [SD] | | 9.1 [0.1] | 9.2 [0.1] | 9.2 [0.1] | 9.2 [1.3] | | 8.7 [2.7] | | 8.9 [2.2] | |
| **PHQ9, n (%)** | |  |  |  |  | |  | |  | |
|  | Minimal depression | 305 (73.7) | 276 (70.6) | 581 (72.2) | 38 (70.4) | | 47 (66.2) | | 85 (68.0) | |
|  | Moderate depression (5-14) | 99 (23.9) | 107 (27.4) | 206 (25.6) | 13 (24.1) | | 20 (28.2) | | 33 (26.4) | |
|  | Moderately severe depression or severe (>14) | 10 (2.4) | 8 (2.1) | 18 (2.2) | 3 (5.6) | | 4 (5.6) | | 7 (5.6) | |
| **Smoking, n (%)** | |  |  |  |  | |  | |  | |
|  | You are Smoker | 16 (3.9) | 11 (2.8) | 27 (3.4) | 0 | | 1 (1.4) | | 1 (0.8) | |
|  | Never smoked | 140 (33.8) | 158 (40.4) | 298 (37.0) | 28 (51.9) | | 27 (38.0) | | 55 (44.0) | |
|  | You were a smoker | 258 (62.3) | 222 (56.8) | 480 (59.6) | 26 (48.2) | | 43 (30.6) | | 69 (55.2) | |
| Body mass index (BMI), mean [SD] | | 27.9 [0.2] | 27.3 [0.2] | 27.6 [0.2] | 28.2 [4.6] | | 27.1 [4.48] | | 27.6 [4.6] | |
| Low density lipoprotein (LDL), mean [SD] | | 88.1 [1.8] | 88.1 [1.3] | 88.1 [1.3] | 89.7 [41.6] | | 91 [40.9] | | 90.4 [41.0] | |
| Systolic blood pressure (SBP), mean [SD] | | 128 [1.0] | 129 [1.0] | 129 [0.7] | 130 [18.7] | | 133 [22.1] | | 132 [20.6] | |
| Diastolic blood pressure (DBP), mean [SD] | | 72 [0.6] | 72 [0.6] | 72 [0.4] | 72 [11.2] | | 73 [11.9] | | 72 [11.5] | |
| Heart rate (HR), mean [SD] | | 69 [0.6] | 68 [0.5] | 69 [0.4] | 71 [11.8] | | 70 [10.9] | | 71 [11.3] | |
| Thromboxane B2 (TBX2), mean [SD] | | 61.2 [6.6] | 58.8 [7.0] | 60.0 [4.8] | 87 [228]^a^ | | 94 [278.5] ^a^ | | 91.0 [257]^b^ | |
| (a) N-1 due to lack of urine sample at the time of the first visit. (b) N-2 | | | |  |  |  | |  | |  |
